# Supplementary material for: HER2-Low Breast Cancer at the Interface of Pathology and Technology: Toward Precision Management
Source: Biomedicines. 2025 Dec 25;14(1):49. doi: 10.3390/biomedicines14010049 (PMC12838288; doi:10.3390/biomedicines14010049)
Supplement: Supplementary file 1 [file biomedicines-14-00049-s001.zip › biomedicines-4027126-supplementary.pdf]

HER2-Low Breast Cancer at the Interface of Pathology and Technology: Toward Precision Management

Faezeh Shekari[1†], Reza Bayat Mokhtari[1,2,3,4†*], Razieh Salahandish[5,6], Manpreet Sambi[1], Roshanak Tarrahi[1], Mahsa Salehi[1], Neda Ashaeri[1] , Paige Eversole[2], Myron Szewczuk[1], Sayan Chakraborty[2], Narges Baluch[7*]

[1]Department of Biomedical and Molecular Sciences, Queen's University, Kingston, ON K7L 3N6, Canada
[2]Department of Pharmacology and Therapeutics, Roswell Park Comprehensive Cancer Center, Buffalo, NY14263, USA
[3]Association of Clinical Immunology and Cancer Research, San Diego, CA, 92127, USA
[4] California Comprehensive Allergy and Food Institute, P.C., San Diego, CA, 92131, USA
[5]Laboratory of Advanced Biotechnologies for Health Assessments (Lab-HA), Biomedical Engineering Program, Lassonde School of Engineering, York University, Toronto, ON, M3J 1P3, Canada
[6]Department of Electrical Engineering and Computer Science (EECS), Lassonde School of Engineering, York University, Toronto, ON, M3J 1P3, Canada
[7]Department of Immunology and Allergy, Rady Children's Hospital, San Diego, CA 92123, USA

* Equivalent Correspondences: cacrbm@gmail.com; nbaluch@rchsd.org
† Equivalent first contributing authors.

Table S1. A list of clinical trials reported results for HER2-low breast cancer patients.

Class	Antibody	Drug Conjugated to the antibody	Clinical Trial Registration Number	Phase of the Trial	Recruiting Status (Dec 2024)	Outcome Assessment
ADCs	Anti-HER2 (Trastuzumab)	Duocarmazine (SYD985)	NCT02277717	Phase 1	Completed	Demonstrated the safety, tolerability, and preliminary efficacy in heavily pretreated patients [1]
	Anti-HER2 (Trastuzumab)	Deruxtecan	NCT04494425 (Destiny Breast 06)	Phase 3	Active, not recruiting	Improved PFS in HER2-low mBC [2, 3]
	Anti-HER2 (Trastuzumab)	Deruxtecan	NCT04553770	Phase 2	Recruiting	Ongoing [4]
	Anti-HER2 (Trastuzumab)	MMAE	NCT04742153	Phase 2	Unknown status	Promising efficacy, especially in heavily pre-treated HER2-low BC patients, with ORR and DCR comparable to other HER2-targeted ADCs [5]
	Anti-HER2 (Trastuzumab)	MMAE	NCT04400695	Phase 3	Recruiting	-
	Anti-HER2 (Trastuzumab)	Deruxtecan	NCT04556773 (DESTINY-Breast08)	Phase 1b	Active, not recruiting	Combined with Anastrazole demonstrated higher response rates compared to combination with Fulvestrant, suggesting potential differences in efficacy between the combinations [6]
	Anti-HER2 (Trastuzumab)	Deruxtecan	NCT03734029 (DESTINY-Breast04)	Phase 3	Active, not recruiting	Improved PFS and OS in HER2-low metastatic BC [7]
	Anti-HER2 (Trastuzumab)	Deruxtecan	NCT04132960 (DAISY trial)	Phase 2	Active, not recruiting	Patients with HER2-low and even some with HER2 non-expressing tumors experienced clinical benefit, indicating that additional mechanisms beyond HER2 targeting might contribute to the efficacy [8]
	Anti-HER2 (Trastuzumab)	Deruxtecan	NCT04420598 (DEBBRAH Study)	Phase 2	Completed	Demonstrated encouraging efficacy and a manageable safety profile in patients with leptomeningeal carcinomatosis, including both HER2-positive and HER2-low subgroups [9]
	Anti-HER2 (Trastuzumab)	MMAE (MRG002)	NCT04742153	Phase 2	Unknown status	promising efficacy and a manageable safety profile in HER2-low advanced or metastatic BC patients [10]
	Anti-HER2 (BL-B07D1)	A cathepsin B cleavable linker, and a topoisomerase I inhibitor (Ed-04)	NCT05461768	Phase 1	Recruiting	Dose-limiting Toxicity, Maximum Tolerated Dose, Recommended Dose [11, 12]
	Anti-HER2 (AR×788)	Amberstatin 269, a potent cytotoxic tubulin inhibitor.	NCT05018676	Phase 2	Unknown status	-
	Anti–Trop-2	SN-38 Sacituzumab Govitecan	NCT02574455	Phase 3	Completed	Improved PFS, OS, ORR [13, 14]
	Anti–Trop-2	SN-38 Sacituzumab Govitecan	NCT04454437	Phase 2b	Active, not recruiting	similar anti-tumor activity and manageable safety profiles in both HER2-0 and HER2-low subgroups of Chinese mTNBC patients [15]
	Anti–Trop-2	SN-38 Sacituzumab Govitecan	NCT03901339	Phase 3	Completed	Demonstrated safety and improved PFS in HER2-low and HER2-0 [16]
	Anti-HER2	MMAE (RC48)	NCT02881138 NCT03052634	dose-escalation phase I, phase Ib	Completed	Manageable Safety and consistent efficacy across HER2-positive and HER2-low expressing BC subgroups, with the 2.0 mg/kg dose every 2 weeks showing the most favorable benefit-risk ratio [17]

	Anti-PDL1 (Toripalimab)	Amberstatin 269 (ARX788)	CTR20222247	Phase 1	-	acceptable safety profile and promising efficacy for HER2-low [18]
	Anti-HER2	PBD (pyrrolobenzodi azepine) dimer	NCT03451162 (DHES0815A)	Phase 1	Completed	Improved antitumor activity but stopped due to persistent toxicities [19]
**Inhibitors**	HER2 inhibitor (pyrotinib)		NCT05165225 (PILHLE-001)	Phase 2	Active, not recruiting	Encouraging efficacy and acceptable toxicity in patients with HR-positive/HER2-low early breast cancer [20]
	HER2-targeted thorium-227 conjugate (BAY2701439)		NCT04147819	Phase 1	Completed	-
**Vaccine**	Anti-HER2 (Trastuzumab) and nelipepimut-S (NPS) vaccine		NCT01570036	Phase 2	Completed	No significant difference in DFS in HER2-low but significant clinical benefit in patients with TNBC [21].

BC, Breast Cancer; ORR, objective response rate; ADC, antibody-drug conjugates; PFS, progression-free survival; OS, overall survival; DFS, disease-free survival; DCR, disease control rate; MMAE, Monomethyl Auristatin E.

Table S2. An overview of studies analyzed the correlation between tissue HER2 and serum HER2 ECD levels in breast cancer patients.

Number of patients and type of BC	Serum assay	Threshold	Tissue HER2 assessment	Correlation	Year	Ref
**94 primary BC**	ADVIA Centaur	15 ng/mL	IHC, ISH (Novocastra-Leica 2C SPEC ERBB2/CEN 17 Probe Kit)	Yes	2021	[22]
**213 metastatic BC**	ADVIA Centaur	15.2 ng/mL	IHC (anti-HER2/neu antibody, clone number 4B5, VENTANA), ISH (PathVysion probe test)	Yes (stage III and IV)	2020	[23]
**43 primary and metastatic BC**	Nuclea Diagnostic	15 ng/mL	-	No	2019	[24]
**100 primary BC**	ADVIA Centaur	15.2 ng/mL	IHC (The monoclonal antibodies CB11, Novocastra; CBE1 and Tab 250, Zymed)	Yes	2019	[25]
**334 primary and metastatic BC**	ADVIA Centaur	15 ng/mL	IHC (clone Ventana pathway HER2 4B5), ISH (INFORM HER2 dual ISH DNA probe cocktail)	No	2016	[26]
**164 primary BC, 382 advanced stages**	ADVIA Centaur	15 ng/mL	-	Yes	2016	[27]
**64 primary and metastatic BC**	BioVendor	30.5 ng/mL	IHC (clone BV5, Diagnostic Biosystems), ISH (PathVysion HER-2 DNA probe kit)	Yes	2016	[28]
**118 primary BC**	Oncogene Science	15 ng/mL	-	No	2015	[29]
**562 primary and metastatic BC**	ADVIA Centaur	30 ng/mL	IHC, ISH	Yes	2014	[30]
**252 primary and metastatic BC**	ADVIA Centaur	15.2 ng/mL	IHC (Hercep Test kit), ISH (PathVysion HER-2 DNA probe kit)	No	2013	[31]
**540 primary BC**	ADVIA Centaur	15 µg/L	IHC (Hercep Test kit), ISH (pharmDx kit)	Yes (for tissue HER2-positive tumor)	2013	[32]
**232 stage I—III BC**	Bender Medsystem	7.4 ng/mL	IHC (polyclonal rabbit anti-human HER2 oncoprotein, Dako); ISH (PathVysion HER-2 DNA probe kit)	Yes	2012	[33]
**200 primary BC**	ADVIA Centaur	15 ng/mL	IHC, ISH	No	2012	[34]
**252 primary BC**	ADVIA Centaur	15 ng/mL	IHC (Monoclonal rabbit antihuman c-erbB-2, Invitrogen), ISH (PathVysion HER-2 DNA probe kit)	Yes	2011	[35]
**74 primary BC**	Bender MedSystem	18.4 ng/mL	IHC (Anti human c-erb-2 oncoprotein code No. A0485, Dakocytomation)	Yes	2010	[36]
**167 primary BC**	Siemens Healthcare Diagnostics	15 ng/mL	IHC (Hercep Test, DakoCytomation), ISH	Yes	2010	[37]
**862 metastatic BC**	ADVIA Centaur	15 ng/mL	IHC (Hercep Test kit), ISH (HER-2 FISH pharmDx kit)	No	2009	[38]
**116 metastatic BC**	Bayer Immuno1	12.7 ng/mL	IHC (Hercep Test kit)	Yes	2008	[39]
**100 metastatic BC**	Bender MedSystems	15 ng/mL	IHC (Rabbit Monoclonal antibody, Labvision)	Yes	2008	[40]
**256 primary BC**	Oncogene Science & ADVIA Centaur	15 ng/mL	IHC (anti-c-HER2 antibody CB11, Biogenex), ISH (PathVysion HER-2 DNA probe kit)	Yes	2008	[41]

**8 primary and metastatic BC**	ADVIA Centaur	15 ng/mL	IHC (Hercep Test kit)	Yes	2007	[42]
**86 primary BC**	ADVIA Centaur	10.2 µg/L	IHC (Dako Corporation), ISH (PathVysion HER-2 DNA probe kit)	No	2006	[43]
**108 primary BC**	Oncogene Science	1368 HNU/mL	IHC (Hercep Test kit)	No	2006	[44]
**195 metastatic BC**	ADVIA Centaur	37 µg/L	IHC (Anti human c-erb-2 oncoprotein code No. A0485, Dako Corp), ISH (PathVysion HER-2 DNA probe kit)	Yes	2006	[45]
**55 metastatic BC**	Bayer Immuno1	15 ng/mL	IHC (Dako Herceptest, rabbit antihuman HER2/neu polyclonal antibody; CB11, mouse antihuman monoclonal antibody; Ventana Medical Systems Inc), ISH (PathVysion HER-2 DNA probe kit)	Yes	2005	[46]
**157 primary BC**	Bayer Immuno1	15 ng/mL	IHC (a rabbit polyclonal antibody A 0485, Dako; a mouse monoclonal antibody CB11, Zymed), ISH	Yes	2005	[47]
**29 metastatic BC**	Oncogene Science	15 ng/mL	IHC (anti-c-HER2 antibody CB11, Novocastra Laboratories)	Yes	2004	[48]
**355 metastatic BC**	Bayer Diagnostics	20 U/mL	IHC (monoclonal antibody CB11 using capillary-gap technology OptiMax Plus Automated Cell Staining System); ISH (Ventana INFORM kit)	Yes	2001	[49]
**140 primary BC**	Bayer Immuno1	13 ng/mL	Measurement of HER-2 concentration in tissue extract (Bayer Immuno 1)	No	2001	[50]
**20 metastatic BC**	Bayer Immuno1	20 ng/mL	IHC (StreptABComplex)	Yes	2000	[51]
**40 metastatic BC**	Calbiochem	450 fmol/mL	IHC (anti-c-HER2 antibody CB11, Biogenex; and LSAB2 detection kit)	Yes	2000	[52]
**158 primary BC**	Nichirei	5.4 ng/mL	IHC (Nichirei, Murine monoclonal anti-human ErbB-2 antibodies, 6G10 and SV-2-61)	Yes	2000	[53]
**63 BC (stage I-IIIB)**	Nichirei	5.4 ng/mL	Measurement of the level of HER-2 in tissue extract (Nichirei)	Yes	1999	[54]
**42 primary BC, 62 stage IV BC**	Triton Diagnostic	20 U/mL		Yes	1997	[55]
**57 primary and metastatic BC**	Bender MedSystems	20 ng/mL	IHC (AP2IN antibody)	No	1996	[56]
**86 primary BC, 77 metastatic BC**	Ciba Corning	15 U/mL	-	Yes	1996	[57]
**168 primary BC**	Oncogene Science	1600 HNU/mL	IHC, ISH	Yes	1995	[58]
**124 breast tumor tissues**	Corning/Triton diagnostics	24 U/mL	Measurement of HER-2 concentration in tissue extract (Corning/Triton diagnostics)	Yes	1995	[59]
**62 primary BC**	Triton Diagnostic	8 U/mL		No	1994	[60]
**79 patients advanced-stage BC**	Triton Diagnostic	10 U/mL	IHC (Triton Diagnostics)	No	1994	[61]

**55 primary BC**	Triton Diagnostic	20 U/mL	IHC (mouse monoclonal antibody, Triton Diagnostics)	No for stage I/II, yes for stage III/IV	1992	[62]

BC, breast cancer; IHC, immunohistochemistry; ISH, in situ hybridization.

References

1. Banerji, U., et al., *Trastuzumab duocarmazine in locally advanced and metastatic solid tumours and HER2-expressing breast cancer: a phase 1 dose-escalation and dose-expansion study.* The Lancet Oncology, 2019. **20**(8): p. 1124-1135.
2. Curigliano, G., et al., *Trastuzumab deruxtecan (T-DXd) vs physician's choice of chemotherapy (TPC) in patients (pts) with hormone receptor-positive (HR+), human epidermal growth factor receptor 2 (HER2)-low or HER2-ultralow metastatic breast cancer (mBC) with prior endocrine therapy (ET): Primary results from DESTINY-Breast06 (DB-06).* Journal of Clinical Oncology, 2024. **42**(17_suppl): p. LBA1000-LBA1000.
3. Bardia, A., et al., *Trastuzumab Deruxtecan after Endocrine Therapy in Metastatic Breast Cancer.* N Engl J Med, 2024.
4. Hurvitz, S.A., et al., *TRIO-US B-12 TALENT: Phase II neoadjuvant trial evaluating trastuzumab deruxtecan with or without anastrozole for HER2-low, HR+ early-stage breast cancer.* Journal of Clinical Oncology, 2022. **40**(16_suppl): p. TPS623-TPS623.
5. Jiang, Z., et al., *A multiple center, open-label, single-arm, phase II clinical trial of MRG002, an HER2-targeted antibody-drug conjugate, in patients with HER2-low expressing advanced or metastatic breast cancer*. 2022, American Society of Clinical Oncology.
6. Jhaveri, K., et al., *Abstract RF02-03: Trastuzumab deruxtecan (T-DXd) in combination with anastrozole or fulvestrant in patients with HER2-low HR+ advanced/metastatic breast cancer: a Phase 1b, open-label, multicenter, dose-expansion study (DESTINY-Breast08).* Cancer Research, 2024. **84**(9_Supplement): p. RF02-03-RF02-03.
7. Modi, S., et al., *Trastuzumab Deruxtecan in Previously Treated HER2-Low Advanced Breast Cancer.* New England Journal of Medicine, 2022. **387**(1): p. 9-20.
8. Mosele, F., et al., *Trastuzumab deruxtecan in metastatic breast cancer with variable HER2 expression: the phase 2 DAISY trial.* Nat Med, 2023. **29**(8): p. 2110-2120.
9. Batista, M.V., et al., *Abstract PS11-05: Trastuzumab Deruxtecan in patients with HER2[+] or HER2-Low Advanced Breast Cancer and Pathologically Confirmed Leptomeningeal Carcinomatosis: Results from Cohort 5 of the DEBBRAH Study.* Cancer Research, 2024. **84**(9_Supplement): p. PS11-05-PS11-05.
10. Jiang, Z., et al., *A multiple center, open-label, single-arm, phase II clinical trial of MRG002, an HER2-targeted antibody-drug conjugate, in patients with HER2-low expressing advanced or metastatic breast cancer.* Journal of Clinical Oncology, 2022. **40**(16_suppl): p. 1102-1102.
11. Song, E., et al., *Abstract PO2-04-03: BL-M07D1, an antibody-drug conjugate directed to HER2 in patients with locally advanced or metastatic Breast Cancer with HER2-positive/low-expression and other solid tumors: Results from a first-in-human phase 1 study.* Cancer Research, 2024. **84**(9_Supplement): p. PO2-04-03-PO2-04-03.
12. Song, E., et al., *685P BL-M07D1, a HER2 antibody-drug conjugate in subjects with locally advanced or metastatic HER2 expressing breast cancer and other solid tumors.* Annals of Oncology, 2023. **34**: p. S478-S479.
13. Hurvitz, S.A., et al., *168P Sacituzumab govitecan (SG) efficacy in patients with metastatic triple-negative breast cancer (mTNBC) by HER2 immunohistochemistry (IHC) status: Findings from the phase III ASCENT study.* Annals of Oncology, 2022. **33**: p. S200-S201.
14. Bardia, A., et al., *Final Results From the Randomized Phase III ASCENT Clinical Trial in Metastatic Triple-Negative Breast Cancer and Association of Outcomes by Human Epidermal Growth Factor Receptor 2 and Trophoblast Cell Surface Antigen 2 Expression.* Journal of Clinical Oncology, 2024. **42**(15): p. 1738-1744.
15. Xu, B., et al., *22MO Efficacy and safety of sacituzumab govitecan in Chinese patients with metastatic triple-negative breast cancer (mTNBC) by baseline HER2 expression level: Subgroup analysis from a phase IIb trial.* Annals of Oncology, 2022. **33**: p. S1438.
16. Schmid, P., et al., *214MO Sacituzumab govitecan (SG) efficacy in hormone receptor-positive/human epidermal growth factor receptor 2-negative (HR+/HER2–) metastatic breast cancer (MBC) by HER2 immunohistochemistry (IHC) status in the phase III TROPiCS-02 study.* Annals of Oncology, 2022. **33**: p. S635-S636.

17. Wang, J., et al., *RC48-ADC, a HER2-targeting antibody-drug conjugate, in patients with HER2-positive and HER2-low expressing advanced or metastatic breast cancer: A pooled analysis of two studies.* Journal of Clinical Oncology, 2021. **39**(15_suppl): p. 1022-1022.
18. Shao, X., et al., *A phase Ia/b trial of ARX788 combined with toripalimab for HER2-low advanced breast cancer (ABC) and other HER2-expressing solid tumors.* Journal of Clinical Oncology, 2024. **42**(16_suppl): p. e13159-e13159.
19. Lewis, G.D., et al., *The HER2-directed antibody-drug conjugate DHES0815A in advanced and/or metastatic breast cancer: preclinical characterization and phase 1 trial results.* Nat Commun, 2024. **15**(1): p. 466.
20. Xia, Y., et al., *Phase II neoadjuvant pyrotinib combined with epirubicin and cyclophosphamide followed by docetaxel in HER2-low–expressing and HR-positive early or locally advanced breast cancer (PILHLE-001): A single-arm trial.* Journal of Clinical Oncology, 2022. **40**(16_suppl): p. TPS620-TPS620.
21. Clifton, G.T., et al., *Results of a Randomized Phase IIb Trial of Nelipepimut-S + Trastuzumab versus Trastuzumab to Prevent Recurrences in Patients with High-Risk HER2 Low-Expressing Breast Cancer.* Clin Cancer Res, 2020. **26**(11): p. 2515-2523.
22. Mokhtari, M. and M.H. Khosravi, *Utility of Serum HER-2/neu in prediction of tissue HER-2/neu status of primary breast cancer.* Middle East Journal of Cancer, 2021. **12**(4): p. 483-490.
23. Zhang, P., et al., *Monitoring value of serum HER2 as a predictive biomarker in patients with metastatic breast cancer.* Cancer Management and Research, 2020: p. 4667-4675.
24. Morgan, S., et al., *Pilot Study on the Utility of Circulating HER2/Neu Levels in the Serum of Breast Cancer Patients.* Anticancer Research, 2019. **39**(10): p. 5345-5352.
25. Fabricio, A.S., et al., *Shed HER2 surrogacy evaluation in primary breast cancer patients: a study assessing tumor tissue HER2 expression at both extracellular and intracellular levels.* Scandinavian Journal of Clinical and Laboratory Investigation, 2019. **79**(4): p. 260-267.
26. Reix, N., et al., *A prospective study to assess the clinical utility of serum HER2 extracellular domain in breast cancer with HER2 overexpression.* Breast cancer research and treatment, 2016. **160**: p. 249-259.
27. Wang, T., et al., *Meaningful interpretation of serum HER2 ECD levels requires clear patient clinical background, and serves several functions in the efficient management of breast cancer patients.* Clinica Chimica Acta, 2016. **458**: p. 23-29.
28. Shukla, S., et al., *Evaluation of HER2/neu oncoprotein in serum & tissue samples of women with breast cancer.* Indian Journal of Medical Research, 2016. **143**(Suppl 1): p. S52-S58.
29. Tchou, J., et al., *Monitoring serum HER2 levels in breast cancer patients.* Springerplus, 2015. **4**: p. 1-7.
30. Di Gioia, D., et al., *Serum HER2 supports HER2-testing in tissue at the time of primary diagnosis of breast cancer.* Clinica chimica acta, 2014. **430**: p. 86-91.
31. Kontani, K., et al., *Clinical usefulness of human epidermal growth factor receptor-2 extracellular domain as a biomarker for monitoring cancer status and predicting the therapeutic efficacy in breast cancer.* Cancer biology & therapy, 2013. **14**(1): p. 20-28.
32. Sørensen, P.D., et al., *Serum HER-2: sensitivity, specificity, and predictive values for detecting metastatic recurrence in breast cancer patients.* Journal of cancer research and clinical oncology, 2013. **139**: p. 1005-1013.
33. Li, M., et al., *Relationship between serum HER2 extracellular domain levels, tissue HER2 expression, and clinico-pathological parameters in early stage breast cancer.* Chinese Medical Journal, 2012. **125**(22): p. 4104-4110.
34. Ryu, D.W. and C.H. Lee, *Impact of serum HER2 levels on survival and its correlation with clinicopathological parameters in women with breast cancer.* Journal of breast cancer, 2012. **15**(1): p. 71.
35. Kong, Y., et al., *High serum HER2 extracellular domain levels: correlation with a worse disease-free survival and overall survival in primary operable breast cancer patients.* Journal of cancer research and clinical oncology, 2012. **138**: p. 275-284.
36. Farzadnia, M., et al., *Evaluation of HER2/neu oncoprotein in serum and tissue samples of women with breast cancer: correlation with clinicopathological parameters.* The Breast, 2010. **19**(6): p. 489-492.

37. Witzel, I., et al., *Monitoring serum HER2 levels during neoadjuvant trastuzumab treatment within the GeparQuattro trial.* Breast cancer research and treatment, 2010. **123**: p. 437-445.
38. Sørensen, P.D., et al., *Serum HER-2 concentrations for monitoring women with breast cancer in a routine oncology setting.* Clinical chemistry and laboratory medicine, 2009. **47**(9): p. 1117-1123.
39. Garoufali, A., et al., *Extracellular domain of HER2: a useful marker for the initial workup and follow-up of HER2-positive breast cancer.* J BUON, 2008. **13**(3): p. 409-13.
40. James, R., et al., *Evaluation of immunohistochemistry and enzyme linked immunosorbent assay for HER-2/neu expression in breast carcinoma.* Indian Journal of Clinical Biochemistry, 2008. **23**: p. 345-351.
41. Ludovini, V., et al., *Evaluation of serum HER2 extracellular domain in early breast cancer patients: correlation with clinicopathological parameters and survival.* Annals of oncology, 2008. **19**(5): p. 883-890.
42. Asgeirsson, K.S., et al., *Serum epidermal growth factor receptor and HER2 expression in primary and metastatic breast cancer patients.* Breast Cancer Research, 2007. **9**: p. 1-8.
43. Kong, S.-Y., et al., *Serum HER-2 concentration in patients with primary breast cancer.* Journal of clinical pathology, 2006. **59**(4): p. 373-736.
44. Quaranta, M., et al., *c-erbB-2 protein level in tissue and sera of breast cancer patients: a possibly useful clinical correlation.* Tumori Journal, 2006. **92**(4): p. 311-317.
45. Kong, S.-Y., et al., *Predicting tissue HER2 status using serum HER2 levels in patients with metastatic breast cancer.* Clinical chemistry, 2006. **52**(8): p. 1510-1515.
46. Fornier, M., et al., *Serum HER2 extracellular domain in metastatic breast cancer patients treated with weekly trastuzumab and paclitaxel: association with HER2 status by immunohistochemistry and fluorescence in situ hybridization and with response rate.* Annals of Oncology, 2005. **16**(2): p. 234-239.
47. Pallud, C., et al., *Tissue expression and serum levels of the oncoprotein HER-2/neu in 157 primary breast tumours.* Anticancer research, 2005. **25**(2B): p. 1433-1440.
48. Müller, V., et al., *Prognostic and predictive impact of the HER-2/neu extracellular domain (ECD) in the serum of patients treated with chemotherapy for metastatic breast cancer.* Breast cancer research and treatment, 2004. **86**: p. 9-18.
49. Harris, L.N., et al., *Comparison of methods of measuring HER-2 in metastatic breast cancer patients treated with high-dose chemotherapy.* Journal of clinical oncology, 2001. **19**(6): p. 1698-1706.
50. Dittadi, R., et al., *Evaluation of HER-2/neu in serum and tissue of primary and metastatic breast cancer patients using an automated enzyme immunoassay.* The International journal of biological markers, 2001. **16**(4): p. 255-261.
51. Cheung, K., et al., *The role of blood tumor marker measurement (using a biochemical index score and c-erbB2) in directing chemotherapy in metastatic breast cancer.* The International journal of biological markers, 2000. **15**(3): p. 203-209.
52. Colomer, R., et al., *Circulating HER2 extracellular domain and resistance to chemotherapy in advanced breast cancer.* Clinical Cancer Research, 2000. **6**(6): p. 2356-2362.
53. Sugano, K., et al., *Combined measurement of the c-erbb-2 protein in breast carcinoma tissues and sera is useful as a sensitive tumor marker for monitoring tumor relapse.* International journal of cancer, 2000. **89**(4): p. 329-336.
54. Imoto, S., T. Kitoh, and T. Hasebe, *Serum c-erbB-2 levels in monitoring of operable breast cancer patients.* Japanese Journal of clinical oncology, 1999. **29**(7): p. 336-339.
55. Krainer, M., et al., *Tissue expression and serum levels of HER-2/neu in patients with breast cancer.* Oncology, 1997. **54**(6): p. 475-481.
56. Willsher, P.C., et al., *Prognostic significance of serum c-erbB-2 protein in breast cancer patients.* Breast cancer research and treatment, 1996. **40**: p. 251-255.
57. Molina, R., et al., *Serum levels of C-erbB-2 (HER-2/neu) in patients with malignant and non-malignant diseases.* Tumor biology, 1997. **18**(3): p. 188-196.
58. Andersen, T.I., et al., *Detection of C-ERBB-2 related protein in sera from breast cancer patients relationship to ERBB2 gene amplification and c-erbB-2 protein overexpression in tumour.* Acta Oncologica, 1995. **34**(4): p. 499-504.

59. Wu, J.T., et al., *Measurement of c-erbB-2 proteins in sera from patients with carcinomas and in breast tumor tissue cytosols: correlation with serum tumor markers and membrane-bound oncoprotein.* Journal of Clinical Laboratory Analysis, 1995. **9**(3): p. 151-165.
60. Fontana, X., et al., *C-erb-B2 gene amplification and serum level of c-erb-B2 oncoprotein at primary breast cancer diagnosis.* Anticancer research, 1994. **14**(5B): p. 2099-2104.
61. Kandl, H., L. Seymour, and W. Bezwoda, *Soluble c-erbB-2 fragment in serum correlates with disease stage and predicts for shortened survival in patients with early-stage and advanced breast cancer.* British journal of cancer, 1994. **70**(4): p. 739-742.
62. Narita, T., et al., *C-erbB-2 protein in the sera of breast cancer patients.* Breast cancer research and treatment, 1992. **24**: p. 97-102.
